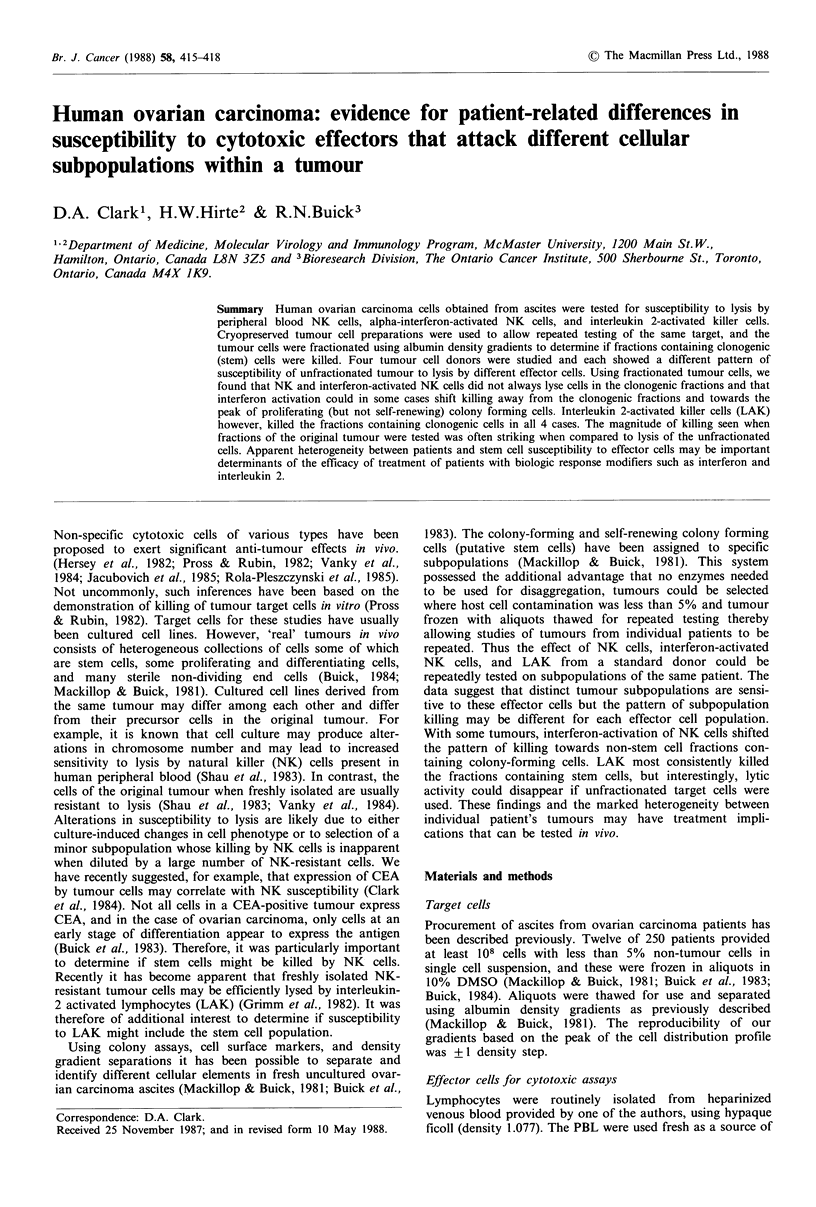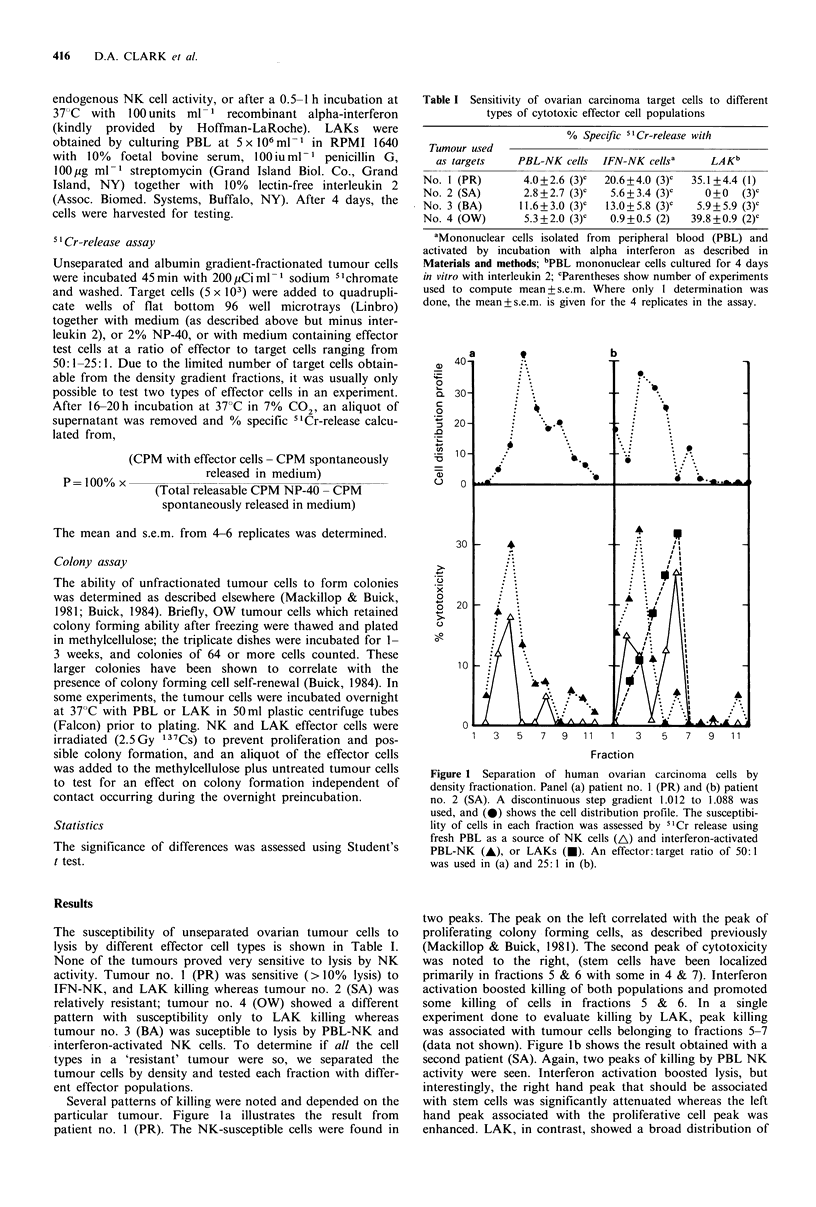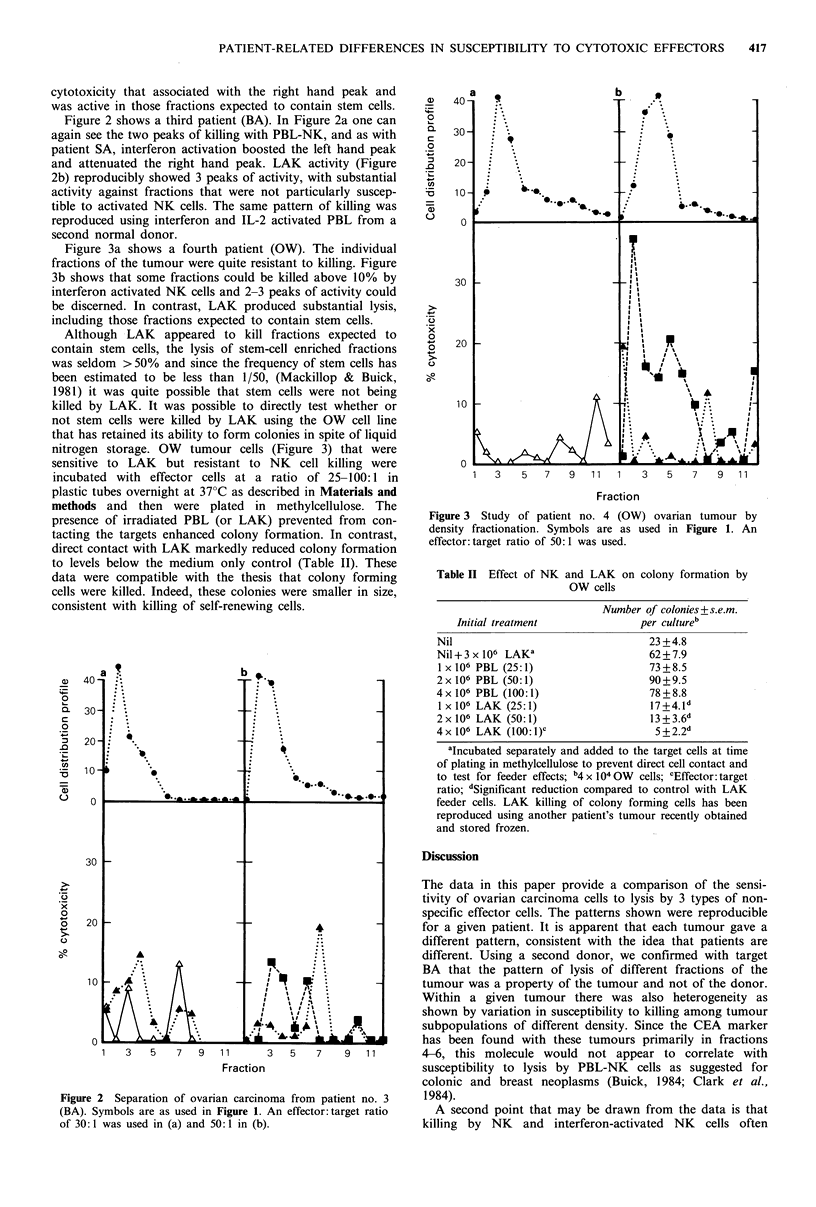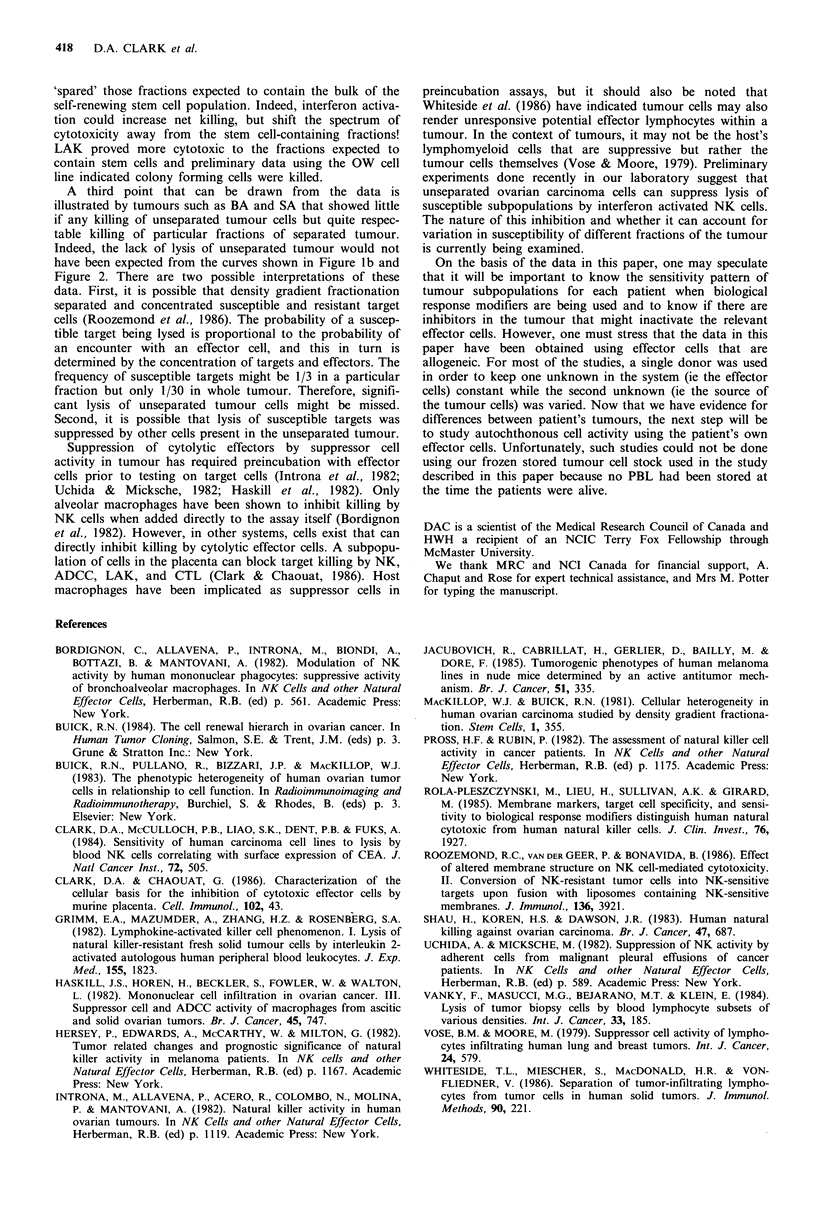# Human ovarian carcinoma: evidence for patient-related differences in susceptibility to cytotoxic effectors that attack different cellular subpopulations within a tumour.

**DOI:** 10.1038/bjc.1988.232

**Published:** 1988-10

**Authors:** D. A. Clark, H. W. Hirte, R. N. Buick

**Affiliations:** Department of Medicine, McMaster University, Hamilton, Ontario, Canada.

## Abstract

Human ovarian carcinoma cells obtained from ascites were tested for susceptibility to lysis by peripheral blood NK cells, alpha-interferon-activated NK cells, and interleukin 2-activated killer cells. Cryopreserved tumour cell preparations were used to allow repeated testing of the same target, and the tumour cells were fractionated using albumin density gradients to determine if fractions containing clonogenic (stem) cells were killed. Four tumour cell donors were studied and each showed a different pattern of susceptibility of unfractionated tumour to lysis by different effector cells. Using fractionated tumour cells, we found that NK and interferon-activated NK cells did not always lyse cells in the clonogenic fractions and that interferon activation could in some cases shift killing away from the clonogenic fractions and towards the peak of proliferating (but not self-renewing) colony forming cells. Interleukin 2-activated killer cells (LAK) however, killed the fractions containing clonogenic cells in all 4 cases. The magnitude of killing seen when fractions of the original tumour were tested were often striking when compared to lysis of the unfractionated cells. Apparent heterogeneity between patients and stem cell susceptibility to effector cells may be important determinants of the efficacy of treatment of patients with biologic response modifiers such as interferon and interleukin 2.


*Br. J. Cancer* (1988) **58**, 415–418 © The Macmillan Press Ltd., 1988

### Human ovarian carcinoma: evidence for patient-related differences in susceptibility to cytotoxic effectors that attack different cellular subpopulations within a tumour

D.A. Clark[1], H.W.Hirte[2] & R.N.Buick[3]

[1,2]*Department of Medicine, Molecular Virology and Immunology Program, McMaster University, 1200 Main St.W., Hamilton, Ontario, Canada L8N 3Z5 and* [3]*Bioresearch Division, The Ontario Cancer Institute, 500 Sherbourne St., Toronto, Ontario, Canada M4X 1K9.*

**Summary** Human ovarian carcinoma cells obtained from ascites were tested for susceptibility to lysis by peripheral blood NK cells, alpha-interferon-activated NK cells, and interleukin 2-activated killer cells. Cryopreserved tumour cell preparations were used to allow repeated testing of the same target, and the tumour cells were fractionated using albumin density gradients to determine if fractions containing clonogenic (stem) cells were killed. Four tumour cell donors were studied and each showed a different pattern of susceptibility of unfractionated tumour to lysis by different effector cells. Using fractionated tumour cells, we found that NK and interferon-activated NK cells did not always lyse cells in the clonogenic fractions and that interferon activation could in some cases shift killing away from the clonogenic fractions and towards the peak of proliferating (but not self-renewing) colony forming cells. Interleukin 2-activated killer cells (LAK) however, killed the fractions containing clonogenic cells in all 4 cases. The magnitude of killing seen when fractions of the original tumour were tested was often striking when compared to lysis of the unfractionated cells. Apparent heterogeneity between patients and stem cell susceptibility to effector cells may be important determinants of the efficacy of treatment of patients with biologic response modifiers such as interferon and interleukin 2.

Non-specific cytotoxic cells of various types have been proposed to exert significant anti-tumour effects *in vivo*. (Hersey *et al.*, 1982; Pross & Rubin, 1982; Vanky *et al.*, 1984; Jacubovich *et al.*, 1985; Rola-Pleszczynski *et al.*, 1985). Not uncommonly, such inferences have been based on the demonstration of killing of tumour target cells *in vitro* (Pross & Rubin, 1982). Target cells for these studies have usually been cultured cell lines. However, 'real' tumours *in vivo* consists of heterogeneous collections of cells some of which are stem cells, some proliferating and differentiating cells, and many sterile non-dividing end cells (Buick, 1984; Mackillop & Buick, 1981). Cultured cell lines derived from the same tumour may differ among each other and differ from their precursor cells in the original tumour. For example, it is known that cell culture may produce alterations in chromosome number and may lead to increased sensitivity to lysis by natural killer (NK) cells present in human peripheral blood (Shau *et al.*, 1983). In contrast, the cells of the original tumour when freshly isolated are usually resistant to lysis (Shau *et al.*, 1983; Vanky *et al.*, 1984). Alterations in susceptibility to lysis are likely due to either culture-induced changes in cell phenotype or to selection of a minor subpopulation whose killing by NK cells is inapparent when diluted by a large number of NK-resistant cells. We have recently suggested, for example, that expression of CEA by tumour cells may correlate with NK susceptibility (Clark *et al.*, 1984). Not all cells in a CEA-positive tumour express CEA, and in the case of ovarian carcinoma, only cells at an early stage of differentiation appear to express the antigen (Buick *et al.*, 1983). Therefore, it was particularly important to determine if stem cells might be killed by NK cells. Recently it has become apparent that freshly isolated NK-resistant tumour cells may be efficiently lysed by interleukin-2 activated lymphocytes (LAK) (Grimm *et al.*, 1982). It was therefore of additional interest to determine if susceptibility to LAK might include the stem cell population.

Using colony assays, cell surface markers, and density gradient separations it has been possible to separate and identify different cellular elements in fresh uncultured ovarian carcinoma ascites (Mackillop & Buick, 1981; Buick *et al.*, 1983). The colony-forming and self-renewing colony forming cells (putative stem cells) have been assigned to specific subpopulations (Mackillop & Buick, 1981). This system possessed the additional advantage that no enzymes needed to be used for disaggregation, tumours could be selected where host cell contamination was less than 5% and tumour frozen with aliquots thawed for repeated testing thereby allowing studies of tumours from individual patients to be repeated. Thus the effect of NK cells, interferon-activated NK cells, and LAK from a standard donor could be repeatedly tested on subpopulations of the same patient. The data suggest that distinct tumour subpopulations are sensitive to these effector cells but the pattern of subpopulation killing may be different for each effector cell population. With some tumours, interferon-activation of NK cells shifted the pattern of killing towards non-stem cell fractions containing colony-forming cells. LAK most consistently killed the fractions containing stem cells, but interestingly, lytic activity could disappear if unfractionated target cells were used. These findings and the marked heterogeneity between individual patient's tumours may have treatment implications that can be tested *in vivo*.

### Materials and methods

### *Target cells*

Procurement of ascites from ovarian carcinoma patients has been described previously. Twelve of 250 patients provided at least $10^8$ cells with less than 5% non-tumour cells in single cell suspension, and these were frozen in aliquots in 10% DMSO (Mackillop & Buick, 1981; Buick *et al.*, 1983; Buick, 1984). Aliquots were thawed for use and separated using albumin density gradients as previously described (Mackillop & Buick, 1981). The reproducibility of our gradients based on the peak of the cell distribution profile was $\pm 1$ density step.

### *Effector cells for cytotoxic assays*

Lymphocytes were routinely isolated from heparinized venous blood provided by one of the authors, using hypaque ficoll (density 1.077). The PBL were used fresh as a source of

Correspondence: D.A. Clark.
Received 25 November 1987; and in revised form 10 May 1988.

endogenous NK cell activity, or after a 0.5–1 h incubation at 37°C with 100 units $ml^{-1}$ recombinant alpha-interferon (kindly provided by Hoffman-LaRoche). LAKs were obtained by culturing PBL at $5 \times 10^6 ml^{-1}$ in RPMI 1640 with 10% foetal bovine serum, 100 iu $ml^{-1}$ penicillin G, 100 $\mu g$ $ml^{-1}$ streptomycin (Grand Island Biol. Co., Grand Island, NY) together with 10% lectin-free interleukin 2 (Assoc. Biomed. Systems, Buffalo, NY). After 4 days, the cells were harvested for testing.

*$^{51}Cr$-release assay*

Unseparated and albumin gradient-fractionated tumour cells were incubated 45 min with 200 $\mu Ci$ $ml^{-1}$ sodium $^{51}$chromate and washed. Target cells ($5 \times 10^3$) were added to quadruplicate wells of flat bottom 96 well microtrays (Linbro) together with medium (as described above but minus interleukin 2), or 2% NP-40, or with medium containing effector test cells at a ratio of effector to target cells ranging from 50:1–25:1. Due to the limited number of target cells obtainable from the density gradient fractions, it was usually only possible to test two types of effector cells in an experiment. After 16–20 h incubation at 37°C in 7% $CO_2$, an aliquot of supernatant was removed and % specific $^{51}$Cr-release calculated from,

$$P = 100\% \times \frac{\text{(CPM with effector cells − CPM spontaneously released in medium)}}{\text{(Total releasable CPM NP-40 − CPM spontaneously released in medium)}}$$

The mean and s.e.m. from 4–6 replicates was determined.

*Colony assay*

The ability of unfractionated tumour cells to form colonies was determined as described elsewhere (Mackillop & Buick, 1981; Buick, 1984). Briefly, OW tumour cells which retained colony forming ability after freezing were thawed and plated in methylcellulose; the triplicate dishes were incubated for 1–3 weeks, and colonies of 64 or more cells counted. These larger colonies have been shown to correlate with the presence of colony forming cell self-renewal (Buick, 1984). In some experiments, the tumour cells were incubated overnight at 37°C with PBL or LAK in 50 ml plastic centrifuge tubes (Falcon) prior to plating. NK and LAK effector cells were irradiated (2.5 Gy $^{137}Cs$) to prevent proliferation and possible colony formation, and an aliquot of the effector cells was added to the methylcellulose plus untreated tumour cells to test for an effect on colony formation independent of contact occurring during the overnight preincubation.

*Statistics*

The significance of differences was assessed using Student's *t* test.

**Table I** Sensitivity of ovarian carcinoma target cells to different types of cytotoxic effector cell populations

| *Tumour used as targets* | *% Specific $^{51}Cr$-release with* | | |
|---|---|---|---|
| | *PBL-NK cells* | *IFN-NK cells*[a] | *LAK*[b] |
| No. 1 (PR) | 4.0±2.6 (3)[c] | 20.6±4.0 (3)[c] | 35.1±4.4 (1) |
| No. 2 (SA) | 2.8±2.7 (3)[c] | 5.6±3.4 (3)[c] | 0±0 (3)[c] |
| No. 3 (BA) | 11.6±3.0 (3)[c] | 13.0±5.8 (3)[c] | 5.9±5.9 (3)[c] |
| No. 4 (OW) | 5.3±2.0 (3)[c] | 0.9±0.5 (2) | 39.8±0.9 (2)[c] |

[a]Mononuclear cells isolated from peripheral blood (PBL) and activated by incubation with alpha interferon as described in **Materials and methods**; [b]PBL mononuclear cells cultured for 4 days *in vitro* with interleukin 2; [c]Parentheses show number of experiments used to compute mean±s.e.m. Where only 1 determination was done, the mean±s.e.m. is given for the 4 replicates in the assay.

**Figure 1** Separation of human ovarian carcinoma cells by density fractionation. Panel (a) patient no. 1 (PR) and (b) patient no. 2 (SA). A discontinuous step gradient 1.012 to 1.088 was used, and (●) shows the cell distribution profile. The susceptibility of cells in each fraction was assessed by $^{51}$Cr release using fresh PBL as a source of NK cells (△) and interferon-activated PBL-NK (▲), or LAKs (■). An effector:target ratio of 50:1 was used in (a) and 25:1 in (b).

### Results

The susceptibility of unseparated ovarian tumour cells to lysis by different effector cell types is shown in Table I. None of the tumours proved very sensitive to lysis by NK activity. Tumour no. 1 (PR) was sensitive (>10% lysis) to IFN-NK, and LAK killing whereas tumour no. 2 (SA) was relatively resistant; tumour no. 4 (OW) showed a different pattern with susceptibility only to LAK killing whereas tumour no. 3 (BA) was suceptible to lysis by PBL-NK and interferon-activated NK cells. To determine if *all* the cell types in a 'resistant' tumour were so, we separated the tumour cells by density and tested each fraction with different effector populations.

Several patterns of killing were noted and depended on the particular tumour. Figure 1a illustrates the result from patient no. 1 (PR). The NK-susceptible cells were found in two peaks. The peak on the left correlated with the peak of proliferating colony forming cells, as described previously (Mackillop & Buick, 1981). The second peak of cytotoxicity was noted to the right, (stem cells have been localized primarily in fractions 5 & 6 with some in 4 & 7). Interferon activation boosted killing of both populations and promoted some killing of cells in fractions 5 & 6. In a single experiment done to evaluate killing by LAK, peak killing was associated with tumour cells belonging to fractions 5–7 (data not shown). Figure 1b shows the result obtained with a second patient (SA). Again, two peaks of killing by PBL NK activity were seen. Interferon activation boosted lysis, but interestingly, the right hand peak that should be associated with stem cells was significantly attenuated whereas the left hand peak associated with the proliferative cell peak was enhanced. LAK, in contrast, showed a broad distribution of cytotoxicity that associated with the right hand peak and was active in those fractions expected to contain stem cells.

Figure 2 shows a third patient (BA). In Figure 2a one can again see the two peaks of killing with PBL-NK, and as with patient SA, interferon activation boosted the left hand peak and attenuated the right hand peak. LAK activity (Figure 2b) reproducibly showed 3 peaks of activity, with substantial activity against fractions that were not particularly susceptible to activated NK cells. The same pattern of killing was reproduced using interferon and IL-2 activated PBL from a second normal donor.

Figure 3a shows a fourth patient (OW). The individual fractions of the tumour were quite resistant to killing. Figure 3b shows that some fractions could be killed above 10% by interferon activated NK cells and 2–3 peaks of activity could be discerned. In contrast, LAK produced substantial lysis, including those fractions expected to contain stem cells.

Although LAK appeared to kill fractions expected to contain stem cells, the lysis of stem-cell enriched fractions was seldom >50% and since the frequency of stem cells has been estimated to be less than 1/50, (Mackillop & Buick, 1981) it was quite possible that stem cells were not being killed by LAK. It was possible to directly test whether or not stem cells were killed by LAK using the OW cell line that has retained its ability to form colonies in spite of liquid nitrogen storage. OW tumour cells (Figure 3) that were sensitive to LAK but resistant to NK cell killing were incubated with effector cells at a ratio of 25–100:1 in plastic tubes overnight at 37°C as described in **Materials and methods** and then were plated in methylcellulose. The presence of irradiated PBL (or LAK) prevented from contacting the targets enhanced colony formation. In contrast, direct contact with LAK markedly reduced colony formation to levels below the medium only control (Table II). These data were compatible with the thesis that colony forming cells were killed. Indeed, these colonies were smaller in size, consistent with killing of self-renewing cells.

**Figure 2** Separation of ovarian carcinoma from patient no. 3 (BA). Symbols are as used in **Figure 1**. An effector: target ratio of 30:1 was used in (a) and 50:1 in (b).

**Figure 3** Study of patient no. 4 (OW) ovarian tumour by density fractionation. Symbols are as used in **Figure 1**. An effector: target ratio of 50:1 was used.

**Table II** Effect of NK and LAK on colony formation by OW cells

| *Initial treatment* | *Number of colonies* ± *s.e.m. per culture*[b] |
|---|---|
| Nil | 23±4.8 |
| Nil+3×10⁶ LAK[a] | 62±7.9 |
| 1×10⁶ PBL (25:1) | 73±8.5 |
| 2×10⁶ PBL (50:1) | 90±9.5 |
| 4×10⁶ PBL (100:1) | 78±8.8 |
| 1×10⁶ LAK (25:1) | 17±4.1[d] |
| 2×10⁶ LAK (50:1) | 13±3.6[d] |
| 4×10⁶ LAK (100:1)[c] | 5±2.2[d] |

[a]Incubated separately and added to the target cells at time of plating in methylcellulose to prevent direct cell contact and to test for feeder effects; [b]$4\times10^4$ OW cells; [c]Effector: target ratio; [d]Significant reduction compared to control with LAK feeder cells. LAK killing of colony forming cells has been reproduced using another patient's tumour recently obtained and stored frozen.

### Discussion

The data in this paper provide a comparison of the sensitivity of ovarian carcinoma cells to lysis by 3 types of nonspecific effector cells. The patterns shown were reproducible for a given patient. It is apparent that each tumour gave a different pattern, consistent with the idea that patients are different. Using a second donor, we confirmed with target BA that the pattern of lysis of different fractions of the tumour was a property of the tumour and not of the donor. Within a given tumour there was also heterogeneity as shown by variation in susceptibility to killing among tumour subpopulations of different density. Since the CEA marker has been found with these tumours primarily in fractions 4–6, this molecule would not appear to correlate with susceptibility to lysis by PBL-NK cells as suggested for colonic and breast neoplasms (Buick, 1984; Clark *et al.*, 1984).

A second point that may be drawn from the data is that killing by NK and interferon-activated NK cells often

'spared' those fractions expected to contain the bulk of the self-renewing stem cell population. Indeed, interferon activation could increase net killing, but shift the spectrum of cytotoxicity away from the stem cell-containing fractions! LAK proved more cytotoxic to the fractions expected to contain stem cells and preliminary data using the OW cell line indicated colony forming cells were killed.

A third point that can be drawn from the data is illustrated by tumours such as BA and SA that showed little if any killing of unseparated tumour cells but quite respectable killing of particular fractions of separated tumour. Indeed, the lack of lysis of unseparated tumour would not have been expected from the curves shown in Figure 1b and Figure 2. There are two possible interpretations of these data. First, it is possible that density gradient fractionation separated and concentrated susceptible and resistant target cells (Roozemond *et al.*, 1986). The probability of a susceptible target being lysed is proportional to the probability of an encounter with an effector cell, and this in turn is determined by the concentration of targets and effectors. The frequency of susceptible targets might be 1/3 in a particular fraction but only 1/30 in whole tumour. Therefore, significant lysis of unseparated tumour cells might be missed. Second, it is possible that lysis of susceptible targets was suppressed by other cells present in the unseparated tumour.

Suppression of cytolytic effectors by suppressor cell activity in tumour has required preincubation with effector cells prior to testing on target cells (Introna *et al.*, 1982; Uchida & Micksche, 1982; Haskill *et al.*, 1982). Only alveolar macrophages have been shown to inhibit killing by NK cells when added directly to the assay itself (Bordignon *et al.*, 1982). However, in other systems, cells exist that can directly inhibit killing by cytolytic effector cells. A subpopulation of cells in the placenta can block target killing by NK, ADCC, LAK, and CTL (Clark & Chaouat, 1986). Host macrophages have been implicated as suppressor cells in preincubation assays, but it should also be noted that Whiteside *et al.* (1986) have indicated tumour cells may also render unresponsive potential effector lymphocytes within a tumour. In the context of tumours, it may not be the host's lymphomyeloid cells that are suppressive but rather the tumour cells themselves (Vose & Moore, 1979). Preliminary experiments done recently in our laboratory suggest that unseparated ovarian carcinoma cells can suppress lysis of susceptible subpopulations by interferon activated NK cells. The nature of this inhibition and whether it can account for variation in susceptibility of different fractions of the tumour is currently being examined.

On the basis of the data in this paper, one may speculate that it will be important to know the sensitivity pattern of tumour subpopulations for each patient when biological response modifiers are being used and to know if there are inhibitors in the tumour that might inactivate the relevant effector cells. However, one must stress that the data in this paper have been obtained using effector cells that are allogeneic. For most of the studies, a single donor was used in order to keep one unknown in the system (ie the effector cells) constant while the second unknown (ie the source of the tumour cells) was varied. Now that we have evidence for differences between patient's tumours, the next step will be to study autochthonous cell activity using the patient's own effector cells. Unfortunately, such studies could not be done using our frozen stored tumour cell stock used in the study described in this paper because no PBL had been stored at the time the patients were alive.

DAC is a scientist of the Medical Research Council of Canada and HWH a recipient of an NCIC Terry Fox Fellowship through McMaster University.

We thank MRC and NCI Canada for financial support, A. Chaput and Rose for expert technical assistance, and Mrs M. Potter for typing the manuscript.

### References

BORDIGNON, C., ALLAVENA, P., INTRONA, M., BIONDI, A., BOTTAZI, B. & MANTOVANI, A. (1982). Modulation of NK activity by human mononuclear phagocytes: suppressive activity of bronchoalveolar macrophages. In *NK Cells and other Natural Effector Cells*, Herberman, R.B. (ed) p. 561. Academic Press: New York.

BUICK, R.N. (1984). The cell renewal hierarch in ovarian cancer. In *Human Tumor Cloning*, Salmon, S.E. & Trent, J.M. (eds) p. 3. Grune & Stratton Inc.: New York.

BUICK, R.N., PULLANO, R., BIZZARI, J.P. & MACKILLOP, W.J. (1983). The phenotypic heterogeneity of human ovarian tumor cells in relationship to cell function. In *Radioimmunoimaging and Radioimmunotherapy*, Burchiel, S. & Rhodes, B. (eds) p. 3. Elsevier: New York.

CLARK, D.A., MCCULLOCH, P.B., LIAO, S.K., DENT, P.B. & FUKS, A. (1984). Sensitivity of human carcinoma cell lines to lysis by blood NK cells correlating with surface expression of CEA. *J. Natl Cancer Inst.*, **72**, 505.

CLARK, D.A. & CHAOUAT, G. (1986). Characterization of the cellular basis for the inhibition of cytotoxic effector cells by murine placenta. *Cell. Immunol.*, **102**, 43.

GRIMM, E.A., MAZUMDER, A., ZHANG, H.Z. & ROSENBERG, S.A. (1982). Lymphokine-activated killer cell phenomenon. I. Lysis of natural killer-resistant fresh solid tumour cells by interleukin 2-activated autologous human peripheral blood leukocytes. *J. Exp. Med.*, **155**, 1823.

HASKILL, J.S., HOREN, H., BECKLER, S., FOWLER, W. & WALTON, L. (1982). Mononuclear cell infiltration in ovarian cancer. III. Suppressor cell and ADCC activity of macrophages from ascitic and solid ovarian tumors. *Br. J. Cancer*, **45**, 747.

HERSEY, P., EDWARDS, A., MCCARTHY, W. & MILTON, G. (1982). Tumor related changes and prognostic significance of natural killer activity in melanoma patients. In *NK cells and other Natural Effector Cells*, Herberman, R.B. (ed) p. 1167. Academic Press: New York.

INTRONA, M., ALLAVENA, P., ACERO, R., COLOMBO, N., MOLINA, P. & MANTOVANI, A. (1982). Natural killer activity in human ovarian tumours. In *NK Cells and other Natural Effector Cells*, Herberman, R.B. (ed) p. 1119. Academic Press: New York.

JACUBOVICH, R., CABRILLAT, H., GERLIER, D., BAILLY, M. & DORE, F. (1985). Tumorogenic phenotypes of human melanoma lines in nude mice determined by an active antitumor mechanism. *Br. J. Cancer*, **51**, 335.

MACKILLOP, W.J. & BUICK, R.N. (1981). Cellular heterogeneity in human ovarian carcinoma studied by density gradient fractionation. *Stem Cells*, **1**, 355.

PROSS, H.F. & RUBIN, P. (1982). The assessment of natural killer cell activity in cancer patients. In *NK Cells and other Natural Effector Cells*, Herberman, R.B. (ed) p. 1175. Academic Press: New York.

ROLA-PLESZCZYNSKI, M., LIEU, H., SULLIVAN, A.K. & GIRARD, M. (1985). Membrane markers, target cell specificity, and sensitivity to biological response modifiers distinguish human natural cytotoxic from human natural killer cells. *J. Clin. Invest.*, **76**, 1927.

ROOZEMOND, R.C., VAN DER GEER, P. & BONAVIDA, B. (1986). Effect of altered membrane structure on NK cell-mediated cytotoxicity. II. Conversion of NK-resistant tumor cells into NK-sensitive targets upon fusion with liposomes containing NK-sensitive membranes. *J. Immunol.*, **136**, 3921.

SHAU, H., KOREN, H.S. & DAWSON, J.R. (1983). Human natural killing against ovarian carcinoma. *Br. J. Cancer*, **47**, 687.

UCHIDA, A. & MICKSCHE, M. (1982). Suppression of NK activity by adherent cells from malignant pleural effusions of cancer patients. In *NK Cells and other Natural Effector Cells*, Herberman, R.B. (ed) p. 589. Academic Press: New York.

VANKY, F., MASUCCI, M.G., BEJARANO, M.T. & KLEIN, E. (1984). Lysis of tumor biopsy cells by blood lymphocyte subsets of various densities. *Int. J. Cancer*, **33**, 185.

VOSE, B.M. & MOORE, M. (1979). Suppressor cell activity of lymphocytes infiltrating human lung and breast tumors. *Int. J. Cancer*, **24**, 579.

WHITESIDE, T.L., MIESCHER, S., MACDONALD, H.R. & VON-FLIEDNER, V. (1986). Separation of tumor-infiltrating lymphocytes from tumor cells in human solid tumors. *J. Immunol. Methods*, **90**, 221.